# Nurse Participation in Colonoscopy Observation versus the Colonoscopist Alone for Polyp and Adenoma Detection: A Meta-Analysis of Randomized, Controlled Trials

**DOI:** 10.1155/2016/7631981

**Published:** 2015-12-29

**Authors:** Lei Xu, Yu Zhang, Haojun Song, Weihong Wang, Sijie Zhang, Xiaoyun Ding

**Affiliations:** ^1^Department of Gastroenterology, Ningbo No. 1 Hospital, Ningbo 315010, China; ^2^College of Medicine, Ningbo University, Ningbo 315211, China

## Abstract

The role of nurse participation (NP) in colonoscopy observation for polyp and adenoma detection is unclear. This study aimed to evaluate whether nurse participation can improve polyp and adenoma detection. *Patients and Methods*. The PUBMED, EMBASE, and Cochrane Library databases were searched for randomized controlled trials (RCTs) published in English. The outcome measurements included (1) the polyp and adenoma detection rate (PDR and ADR); (2) the advanced lesions detection rate; and (3) the mean polyp and adenoma detection rate per colonoscopy. *Results*. Three RCTs with a total of 1676 patients were included. The pooled data showed a significantly higher ADR in the NP group than colonoscopist alone (CA) (45.7% versus 39.3%; RR 1.16; 95% CI, 1.04–1.30). And it showed no significant difference in the PDR and advanced lesions detection rate between the two groups (RR: 1.14, 95% CI: 0.95–1.37; RR: 1.35, 95% CI: 0.91–2.00; resp.). *Conclusions*. Nurse participation during a colonoscopy can improve the ADR, whereas no benefit for the PDR and advanced lesions detection rate was observed. All RCTs included in the meta-analysis had high risk of bias. Thus, there is a need for new research that uses sound methodology to definitively address the research question under study.

## 1. Introduction

Colorectal cancer (CRC) is one of the most common cancers worldwide. Colonoscopy screening and removal of adenomas are considered the most effective method for reducing the incidence and mortality of CRC [1, 2]. However, the miss rate of colonoscopy screening is as high as 11% for advanced adenomas and 26% for all adenomas [3]. The adenoma detection rate (ADR) and polyp detection rate (PDR), defined as the proportion of colonoscopies in which one or more adenomas (or polyp) are detected, are both considered as a measure for colonoscopy [4, 5]. Moreover, the adenoma detection rate (ADR) is associated with the risks of interval CRC and fatal interval cancer [4, 6]. Several methods and devices have been developed to increase the ADR, including prolonged colonoscopy withdrawal time, improved quality of the bowel preparation, the application of a cap-assisted colonoscopy, and the third eye retroscope [7–9]. However, most of these innovations have not been widely adopted due to the additional workload for the endoscopist or the additional cost for specialized equipment. Investigators have found that participation by an additional observer (fellow, nurse, or trainees) during a colonoscopy may increase the adenoma or polyp detection rate. However, Oh et al. [10] pooled 14 articles and showed that the involvement of a fellow did not affect the adenoma and polyp detection rates. However, this meta-analysis excluded nurse participation, and evidence has shown that nurse participation during a colonoscopy may increase the adenoma or polyp detection rate [11–14].

Here, we performed a meta-analysis of randomized controlled trials (RCTs) to determine whether nurse participation during a colonoscopy can affect the adenoma or polyp detection rate.

## 2. Methods

### 2.1. Inclusion Criteria

We defined the inclusion criteria according to the PICOS [15]: (1) participants (P): all of the patients who received a colonoscopy; (2) interventions (I) and comparisons (C): comparison of nurse participation in the observation (NP) versus the colonoscopist alone (CA) during a colonoscopy; (3) outcomes (O): the primary outcome being the polyp detection rate (PDR) or the adenoma detection rate (ADR), defined as the proportion of patients in which more than one polyp or adenoma was detected. The secondary outcomes included (i) the advanced lesions detection rate, defined as the proportion of patients in which more than one advanced lesion (advanced adenoma [size ≥ 1 cm, villous histology, and high-grade dysplasia], or carcinoma) was detected, (ii) the mean number of polyps per patient, and (iii) the mean number of adenomas per patient; (4) study design (S): randomized controlled trials (RCTs).

### 2.2. Search Strategy

An electronic search was performed using key words combined with medical subject headings (MeSH). We searched full publications and abstracts from the following computerized databases: MEDLINE, EMBASE, and the Cochrane Central Register of Controlled Trials in the Cochrane Library (1981–2014). The key words included “colonoscopy, nurse, polyp, and adenoma”. The search was limited to clinical trials and articles published in English.

### 2.3. Data Extraction

Eligible articles were reviewed independently by two investigators (Lei Xu and Yu Zhang). Discrepancies between the two investigators were resolved by discussion and consensus with a senior investigator (Xiaoyun Ding).

### 2.4. Risk of Bias Assessment

The potential bias was evaluated with the Cochrane Collaboration's tool for assessing risk of bias of RCTs [16]. The risk domains of assessment included (1) random sequence generation; (2) allocation concealment; (3) blinding of participants and personnel; (4) blinding of outcome assessment; (5) incomplete outcome data; (6) selective reporting; and (7) other sources of bias.

### 2.5. Statistical Analysis

Statistical analyses were performed with RevMan software (Review Manager Version 5.2, the Nordic Cochrane Centre, Copenhagen, Denmark). For dichotomous variables compared within each trial, the risk ratios (RR) and 95% confidence intervals (95% CI) were calculated. Statistical heterogeneity among trials was evaluated by Cochrane's *Q* test and a chi-squared test, and a value of *P* < 0.1 was considered significant heterogeneity. The quantity of statistical heterogeneity was assessed with the *I* ^2^ statistic. Due to high values of *I* ^2^, which indicated studies with increased heterogeneity, a random-effect model was applied.

## 3. Results

### 3.1. Selection and Features of Studies

**Figure 1:**
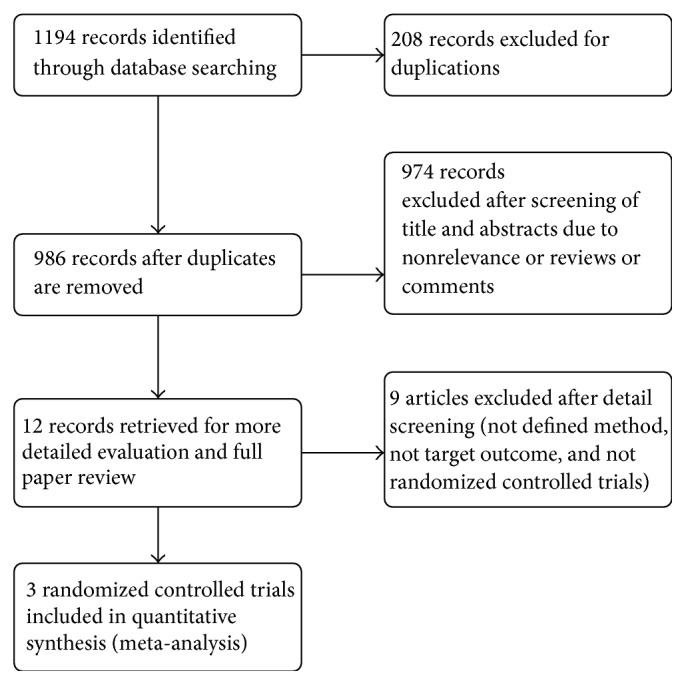
Flow diagram on literature search.

The initial search identified 1194 potential abstracts. The title and abstract were reviewed; 1182 studies were rejected due to duplications, nonrelevance, or the fact that they were reviews or comments. Twelve articles were retrieved for more detailed evaluation and full paper review. Nine articles were excluded due to nonrandomization, nondefined method, and different target outcomes. The final meta-analysis included three studies from 2011 to 2013 [12–14] (Figure 1).

All three studies were published as full articles. The patients all underwent a screening colonoscopy, and the exclusion criteria are listed in Table 1. Two studies were conducted in Korea [12, 13], and one study was conducted in the USA [14]. Two studies were single-center trials [13, 14], while the other study was a multicenter trial [12]. A total of 1676 individuals fulfilled our inclusion criteria in the three trials, and they all underwent a screening colonoscopy.  Table 2 lists the characteristics of the 3 included studies. Overall, 852 patients were randomized to the NP group, and 824 patients were randomized to the NP group. Two of the three studies defined the patient inclusion age as more than 50 years [12, 13]. The average age, proportion of male patients, intubation time, withdrawal time, and adequate bowel preparation rate were similar between the CA and NP groups in individual trials. Two studies mentioned that the nurse used for observation performed along with other in-room duties [12, 14]; one reported that, at any time the nurse was unable to observe the screen, colonoscope withdrawal was paused until the nurse was able to observe the screen again [14].

### 3.2. Risk of Bias

**Figure 2:**
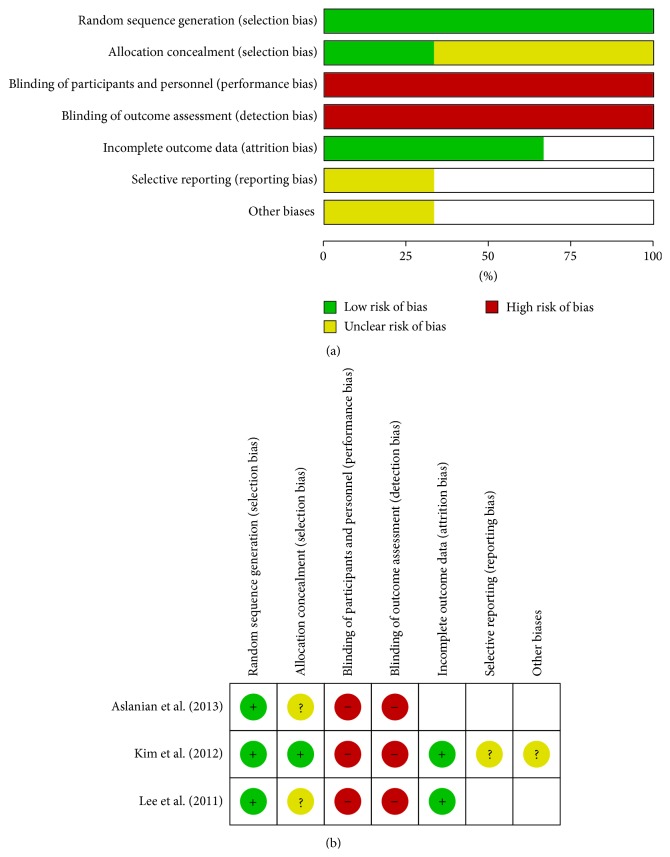
(a) Risk of bias graph. (b) Risk of bias summary.

Among the 3 trials, all studies presented a description of random sequence generation and incomplete outcome data. No study reported the use of blinding and allocation concealment. One of the included studies had a low risk of bias for allocation concealment [13], and another had a low risk of bias for selective reporting [12] (Figure 2).

### 3.3. PDR and ADR

**Figure 3:**
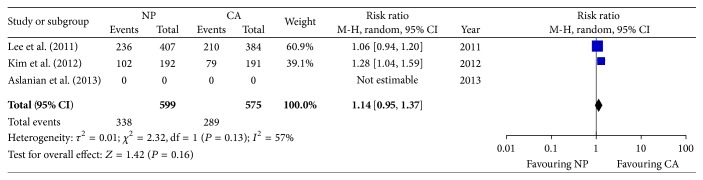
Forest plot on the polyp detection rate comparing NP versus CA. NP: nurse participation in the observation. CA: colonoscopist alone.

The PDR was compared between the NP and CA groups in two trials. Kim et al. [13] showed a significantly increased PDR in the NP group compared to the CA group (53.1% versus 41.3%, adjusted OR: 1.54; 95% CI: 1.00–2.36, *P* < 0.05), while Lee et al. [12] did not show a significant difference in the PDR between the two groups (58.0% (236/407) versus 54.7% (210/384), *P* = 0.350). The pooled data from two RCTs showed no significant difference in the PDR between the NP and CA groups (56.4% versus 50.3%, resp.; RR: 0.95; 95% CI: 0.95–1.37) (Figure 3).

**Figure 4:**
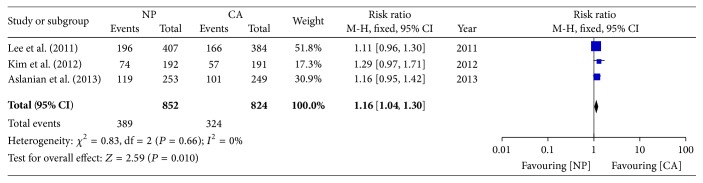
Forest plot on the adenoma detection rate comparing NP versus CA.

The adenoma detection rate was compared in the three trials. Each study showed a trend toward an increased ADR in the NP group compared to the CA group, but no statistically significant difference was achieved. The pooled data from the three trials showed an increased ADR in the NP group compared to the CA group (45.7% versus 39.3%; RR: 1.16; 95% CI: 1.04–1.30) (Figure 4).

### 3.4. Advanced Lesions

**Figure 5:**
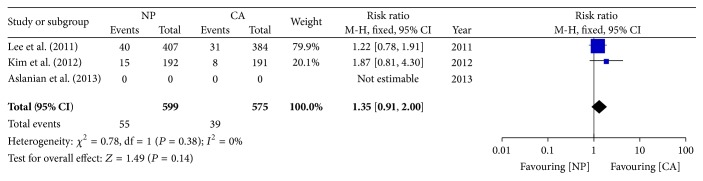
Forest plot on the advanced lesions detection rate comparing NP versus CA.

Two trials [12, 13] showed no significant difference in advanced lesions between the two groups. The pooled data also showed no significant difference (9.2% versus 6.8%; RR: 1.35; 95% CI: 0.91–2.00) (Figure 5).

### 3.5. Mean Polyp and Adenoma Detection per Colonoscopy

All three studies reported the total numbers of polyps and adenomas detected. Lee et al. [12] reported that the mean number of detected polyps per patient (mppp) was significantly higher in the dual-observation group with an experienced nurse than that in the single-observation group (adjusted mean difference: 0.40; 95% CI: 0.03–0.77). The mean number of detected adenomas per patient (mapp) was also significantly higher in the group with a colonoscopist and an experienced nurse than that in the single-observation group (adjusted mean difference: 0.44; 95% CI: 0.05–0.82). Aslanian et al. [14] reported that the mppp and mapp were significantly higher in the dual-observation group than those in the single-observation group (mppp: 1.32 versus 1.03, 95% CI: 1.09–1.51; mapp: 0.82 versus 0.64, 95% CI: 1.038–1.569). Kim et al. [13] reported that the mppp was higher in the dual-observation group (184/192 versus 142/191).

## 4. Discussion

This meta-analysis showed that nurse participation during a colonoscopy improved the colon ADR. There was a tendency toward a higher PDR and advanced lesions when a nurse participated in the observation, although the pooled analysis showed that the differences did not reach statistical significance. Furthermore, the data showed that the mean polyp and adenoma detection rates per colonoscopy were higher when a nurse was involved.

The results of our meta-analysis confirm the expression that “two pairs of eyes are better than one.” Several potential reasons for missing adenomas during a colonoscopy include the following [17]: (1) The polyp was not detected. The polyp may not be present in the field of endoscopic view due to the anatomical location. (2) The polyp was in the field of view but not recognizable. (3) The polyp was recognizable but not detected. The latter indicates that some polyps are within the field of view at the time of the procedure, but the endoscopist did not recognize them. This meta-analysis showed that the addition of a nurse as a second observer could improve the detection of polyps and adenomas. This result is also consistent with studies which have reported that the involvement of a fellow during a colonoscopy improved the adenoma and polyp detection rates [10, 18, 19]. Furthermore, the study by Lee et al. [12] showed that the nurse's experience influenced the PDR and ADR, particularly with an inexperienced colonoscopist. Similar results have shown that the experience of a fellow or trainee may influence the ADR or PDR [19]. Therefore, the participation of well-trained nurses may increase the PDR and ADR during a colonoscopy. Moreover, when no supervisor is present, an endoscopist with less experience may achieve a much higher PDR and ADR by including an experienced nurse.

Smaller polyps are more likely to be missed during a colonoscopy [20]. However, two of the included studies reported that the advanced adenoma detection rate showed an increased trend of detection [12, 13]. As advanced adenomas have a stronger relationship with the colon cancer, more studies are needed to confirm this effect. On the other hand, nonpolypoid depressed adenomas are more difficult to identify during a screening colonoscopy, but they carry a greater risk for developing into high-grade dysplasia or submucosal invasive cancer [21, 22]. The study by Lee et al. [12] reported that only 7 (7/408, 1.7%) nonpolypoid depressed adenomas were found in the dual-observation group, but they did not record whether the nurse or endoscopist found the lesions. More studies are necessary to determine whether dual observation could impact the detection of depressed lesions.

There are some limitations of this meta-analysis. First, only three RCTs were included in this study; more studies are required to assess the role of nurse participation in colonoscopies. Second, variables such as the withdrawal technique, use of narrow band imaging, and high-definition colonoscopy may be associated with the ADR [23–25]. However, only one of the included three studies reported not using chromoendoscopy or other techniques to highlight the colonic mucosa [12]. Two of the included studies reported the use of a high-definition colonoscopy [12, 13]. Third, the study by Lee et al. [12] inspected the colonic mucosa during the withdrawal phase; the study by Aslanian et al. [14] inspected the mucosa during both the insertion and withdrawal phases, while Kim et al. [13] did not report the phase in which inspection occurred. Therefore, nurse observation during the whole examination or only in the withdrawal phase may be a source of heterogeneity. Additionally, our search strategy only included articles published in English. Articles published in other languages were not included due to anticipated difficulties in obtaining accurate medical translations.

## 5. Conclusion

This meta-analysis showed that nurse participation during a colonoscopy can improve the adenoma or polyp detection rate. All RCTs included in the meta-analysis had high risk of bias. Thus, there is a need for new research that uses sound methodology to definitively address the research question under study.

## Figures and Tables

**Table 1:** Characteristics of included randomized controlled trials.

Trials	Country	Colonoscopy indication	Exclusion criteria	*N* (total)	Defined age for patients included	Patients allocated to nurse participation	Patients allocated to colonoscopist alone

Lee et al., 2011 [12]	Korea	Asymptomatic average risk individuals for screening colonoscopy	(1) GI bleeding, history of colorectal surgery, IBD, hereditary colorectal cancer, or polyposis syndrome and inability to provide consent; failed intubation and inadequate withdrawal time	791	≥50	407	384

Kim et al., 2012 [13]	Korea	Average risk patients for screening colonoscopy	(1) Symptoms for lower gastrointestinal tract disease; (2) family history of CRC; (3) personal history of CRC, polyps, or IBD; (4) history of a colorectal examination within 5 years or colorectal surgery; (5) failed to reach the cecum	383	≥50	192	191

Aslanian et al., 2013 [14]	USA	Patients for outpatient screening colonoscopy	IBD, hereditary colorectal cancer syndromes	502	None	253	249

**Table 2:** Characteristics of patients in the included trials.

Trials	Average age (year)		Male/total (%)		Intubation time (min)		Withdrawal time (min)		Bowel preparation (adequate, %)	
	CA	NP	CA	NP	CA	NP	CA	NP	CA	NP
Lee et al., 2011 [12]	58.1 ± 7.3	58.6 ± 7.4	51.3	54.6	7.9 ± 5.9	7.3 ± 4.8	9.7 ± 3.9	10.2 ± 5.5	75.3	76.2
Kim et al., 2012 [13]	56.4 ± 6.1	57.3 ± 6.0	60.2	62.5	7.1 ± 5.7	7.1 ± 5.6	8.5 ± 3.5	8.8 ± 3.5	80.1	78.6
Aslanian et al., 2013 [14]	57.8 ± 9.4	58.1 ± 9.6	53.4	47.4	NS	NS	14.1 ± 8.3	15.3 ± 8.2	88	87.7
